# Mechanical Behaviour of Dental Luting Cements: Static, Dynamic, and Finite Element Studies

**DOI:** 10.3390/dj13120601

**Published:** 2025-12-15

**Authors:** Tamás Tarjányi, Csongor Mészáros, Rebeka Anna Kiss, Zsolt Tóth, István Pelsőczi

**Affiliations:** 1 Department of Medical Physics and Informatics, Faculty of Science and Informatics, Albert Szent-Györgyi Medical School, University of Szeged, Korányi Fasor 9, H-6720 Szeged, Hungary; kissrebeka0125@gmail.com (R.A.K.); ztoth@physx.u-szeged.hu (Z.T.); 2 Department of Operative and Esthetic Dentistry, Faculty of Dentistry, University of Szeged, Tisza Lajos Körút 64-66, H-6720 Szeged, Hungary; csongor021@gmail.com; 3 Department of Prosthodontics, Faculty of Dentistry, University of Szeged, Tisza Lajos Körút 64-66, H-6720 Szeged, Hungary; pelsoczi@stoma.szote.u-szeged.hu

**Keywords:** dental luting cements, static load test, dynamic load test, mechanical stress, compressive strengths

## Abstract

**Background/Objectives:** The long-term clinical success of dental luting cements largely depends on their mechanical performance. This study systematically compared six commonly used definitive dental cements by assessing key mechanical characteristics such as compressive strength and fatigue resistance. **Methods:** The tested materials included Adhesor Zinc Phosphate (AphC), Harvard Zinc Phosphate (HphC), polycarboxylate cement (CaC), glass ionomer cement (GIC), resin-modified glass ionomer cement (RMGIC), and resin cement (ReC). Both static and dynamic compressive load tests were performed using an Instron ElectroPuls E3000 dynamic testing instrument. During static testing, 77 samples were subjected to an increasing load up to 1500 N. Dynamic tests on 78 samples involved cyclic loading over seven phases from 50 N to 1600 N, with 1500 cycles per phase at 10 Hz. **Results:** Static load results indicated that GIC, CaC, and phosphate cements exhibited similar performance and were significantly weaker compared to RMGIC and ReC. In the dynamic fatigue tests, most ReC and RMGIC samples maintained integrity throughout the entire protocol, demonstrating markedly superior mechanical reliability. Finite element analysis (FEA) further confirmed the experimental observations, revealing more homogenous stress distribution and lower peak stresses in ReC and RMGIC compared with the conventional cements. **Conclusions:** Overall, the resin-based and resin-modified glass ionomer cements showed the highest compressive strength and fatigue resistance, indicating superior long-term mechanical stability compared to the conventional cements. These findings support the clinical use of resin-based cements as reliable luting agents for definitive fixation in high-load prosthodontic applications.

## 1. Introduction

Definitive dental luting agents are used in dentistry to retain fixed restorations (e.g., inlays, onlays, crowns, or bridges). The efficacy of cement fixation depends primarily on three factors. Zinc phosphate cement provides a non-adhesive, mechanical retention through a wedge-effect created by the insertion of a luting agent into the interface between two surfaces [1,2]. Resin-based cements establish micro-mechanical anchorage by employing adhesive techniques involving etching and bonding [1,3]. Zinc polycarboxylate and glass ionomer cements (GICs) achieve molecular adhesion through chemical bonding to the tooth structure, significantly increasing the luting effect [1]. The principal setting reactions of dental cements may involve either acid–base reactions or polymerization processes [4].

Selecting an appropriate dental cement in the clinical practice is a complex task, influenced by recent dental material innovations and dependent on several chemical, biological, and physical properties [5]. Key selection factors include solubility, water sorption [6,7], adhesion, biocompatibility, caries or plaque inhibition, microleakage [8,9], mechanical strength along with many mechanical properties [10], wear resistance, radio-opacity, film thickness, viscosity, and working and setting times [11]. Solubility is a relevant chemical property which is mainly influenced by the consistency of the material and the chemical composition of it [6,7]. High solubility contributes greatly to microleakage at the restoration margins [12]. Conventional aqueous-based cements, such as zinc phosphate and zinc polycarboxylate, have higher solubility [11,13]; however, clinical trials have revealed long-term success for restorations cemented with zinc phosphate [14]. In order to decrease the solubility, the recommended powder-to-liquid mixing ratios should be observed and used [11,13]. Ideal cements exhibit minimal water sorption, as excess water uptake compromises mechanical integrity [11]. From the biological perspective, biocompatibility is critical, as cements must not damage the dental pulp and surrounding tissue [8,15]. Low initial setting pH poses major risk factors to the postoperative pulp sensitivity or inflammation [16]. The acidic reaction has a higher risk in cases of thinner dentine walls [14,16,17]. The cavity protective effect gives popularity to the GIC which releases fluoride ions and may potentially decrease caries formation [3,11].

Our investigations focused on the mechanical behaviour of luting cements, as these properties largely determine the long-term durability of a restoration [5,18]. Essential physical properties of dental cements are the compressive strength, tensile strength, and modulus of elasticity (Young’s modulus). These characteristics are critical in determining the long-term success of the fixation by ensuring resistance to mastication forces. Song Chen et al. previously studied the mechanical characteristics of GIC dental cements by fabricating cylinder samples and performed strength and fatigue tests [19]. In their study, they used conventional GIC, zinc-reinforced GIC, RMGIC, and a resin-based composite material. Their results showed that the resin-modified GIC and resin-based composite have superior mechanical properties over the conventional GIC. However, since these results do not cover other widely used dental cements, the analysis was extended to phosphate and carboxylate cements in this study. Furthermore, using the finite element method, the spatial distribution of stress resulting from compressive loading was investigated and its relationship with the strength of different materials was examined.

For proper function over many years, the fixed restoration material has to be sufficiently strong and, as a result, resistant to constant and cyclic forces which may finally result in fatigue fracture of the material. Several in vitro studies have shown that compressive and cyclic loads are among the most critical factors contributing to clinical failure [20,21]. Dynamic fatigue tests effectively simulate the continuous mechanical loading that also occurs normally in the oral cavity due to mastication. For sustained clinical performance, luting materials must withstand compressive and shear forces without structural degradation. Thus, compressive strength and modulus of elasticity are significant indicators of the durability of dental cements [16,19]. The elastic modulus is a physical quantity that characterize the material stiffness, reflecting the required loading force to produce a given elongation or compression [16]. Therefore, a higher modulus value denotes greater stiffness of the material [1,2]. The literature cites a wide range of compressive strengths from 57 MPa to 265 MPa and moduli between 1.2 and 10.7 GPa for different types of cement [11]. Polymerization shrinkage also affects the mechanical behaviour of restorative materials. Some studies have shown that low or non-shrinking materials, such as hybrid glass ionomer cements, generate significantly lower internal mechanical stresses within the bulk [22,23,24].

The purpose of this study was to compare the most commonly used definitive dental luting agents, i.e., dental cements, based on their mechanical properties. Static compressive loading tests were performed to determine and compare the compressive strengths of the different cement types. These strength values were calculated from the measured fracture force and contact area based on the Hertzian model [25,26]. In addition, we simulated the cyclic forces, arising in the oral cavity, with dynamic fatigue loading tests. The results obtained from these tests provide insight into the short- and long-term reliability of the tested cement materials, which have implications for their clinical performance.

## 2. Materials and Methods

### 2.1. Sample Preparation

Standardized cement samples were prepared for the study using custom silicone mould. The following six types of cements were used: Adhesor Zinc Phosphate Cement (AphC, SpofaDental, Jilcin, Czeh Republic), Harvard Zinc Phosphate Cement (HphC, Harvard Dental International GmbH, Hoppegarten, Germany), Harvard Polycarboxylate Cement (CaC, Harvard Dental International GmbH, Hoppegarten, Germany), GC Fuji I CAPSULE Radiopaque Glass Ionomer Luting Cement (GIC, GC Corporation, Tokyo, Japan), GC Fuji PLUS CAPSULE Radiopaque Reinforced Glass Ionomer Luting Cement (RMGIC, GC Corporation, Tokyo, Japan), and G-CEM LinkAce Self Adhesive Resin Cement (ReC, GC Corporation, Tokyo, Japan). The cements were prepared according to the manufacturer’s recommendation.

**Figure 1:**
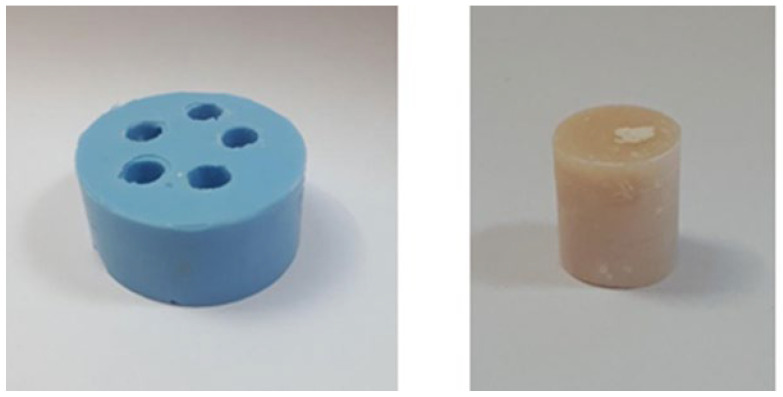
Silicone mould used for preparing standardized cement samples, and a representative RMGIC sample after setting.

The zinc phosphate (AphC and HphC) and polycarboxylate (CaC) cements were in a powder–liquid formulation and a clinician mixed them by hand. The glass ionomers (GIC and RMGIC) and the self-adhesive resin (ReC) cements were in a capsule formulation. All the cements were fabricated at room temperature, and they were inserted in a 0.8 cm diameter silicone mould, which was prepared for the standard cylinder shape of the samples (FeguaSil, Feguramed GmbH, Buchen). The height of the created samples was set to 1 cm (see Figure 1). The samples were stored under laboratory conditions where the temperature was set to 22 °C.

### 2.2. Mechanical Test Protocols

**Figure 2:**
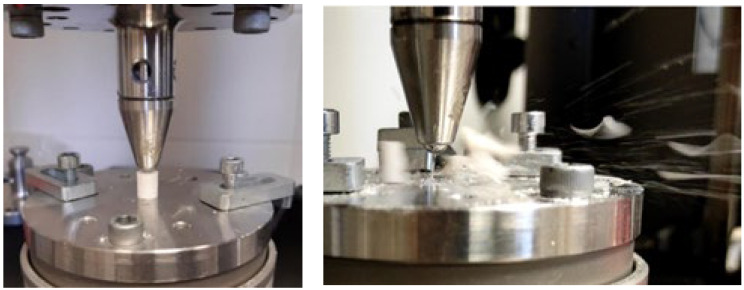
Illustration of the experimental setup for mechanical testing, showing the loading head and a representative dental cement sample (**left**), and a snapshot at the point of fracture (**right**).

Both static and dynamic fatigue loading tests were performed on the samples with the Instron ElectroPuls E3000 electrodynamic instrument (Instron Corporation, Norwood, MA, USA). The samples were subjected to the compressive load by a stainless steel R=2.75 mm sphere that was fixed in a conical loading head (see Figure 2). The spherical loading head provides a clinically relevant approximation of occlusal contact mechanics by replicating how occlusal forces penetrate the cement layer and generate a concentrated subsurface stress zone that promotes microcrack initiation in brittle dental materials. During the static loading tests, a gradually increasing load up to 1500 N was exerted on the samples over 1 min. The total sample number for the static test was n= 77, distributed among the six different groups (12 AphC, 13 HphC, 13 CaC, 13 GIC, 13 RMGIC, 13 ReC). The raw measurements can be found in the Supplementary File S1. From the resulting load–displacement curves, compressive strengths were determined based on the Hertzian contact model [25,26]. With this model, the mechanical stress, induced by a spherical loading head that has a radius of R, can be modelled as it penetrates into a material that has a smooth flat surface. The contact stress can be computed using the following Hertzian formula:

(1) $$\sigma =(1-2\mu_{S})\;\frac{3F}{{2\pi a}^{2}\;}\;,$$

where F is the applied force and a is the contact radius derived from the material properties and geometry of both sample and loading head. According to the Hertzian model, the contact radius is [27,28]

(2) $$a^{3}=\frac{3FR}{4E_{C}}\;,$$

where R is the radius of the spherical loading head and $E_{c}$ is the combined modulus of elasticity. The combined modulus can be calculated based on the modulus of elasticity and Poisson’s ratios of the sample (dental cements) and loading head material (steel):

(3) $$\frac{1}{E_{C}}=\frac{1-\mu_{S}^{2}}{E_{S}}+\frac{1-\mu_{L}^{2}}{E_{L}}\;,$$

where $E_{s}$ and $E_{L}$ are the moduli of elasticity and $\mu_{s}$ and $\mu_{L}$ are the Poisson’s ratios of the sample and loading head, respectively. Table 1 shows the material constants that were used for the calculations based on the literature [10,29,30,31,32]. The material strengths were evaluated by comparing the calculated maximum stress to the failure criteria of each cement type under static loading conditions.

Dynamic fatigue loading tests were carried out to simulate cycling loading conditions. Accelerated dynamic tests involved an incremental loading over seven distinct phases. The maximum load was increased in each phase as follows: 50 N in the first phase, 100 N in the second, 200 N in the third, 400 N in the fourth, 800 N in the fifth, 1200 N in the sixth, and 1600 N in the last phase. Each phase consisted of 1500 cyclic loads applied as a sine wave at a frequency of 10 Hz, resulting in a total of 10,500 cycles on each sample (previously Fráter et al. performed similar fatigue tests [33]). The dynamic test was performed on a total number of n=68 total samples (7 AphC, 11 HphC, 16 CaC, 13 GIC, 11 RMGIC, 10 ReC). All static and fatigue loading tests were conducted at room temperature.

### 2.3. Protocol of the Finite Element Analysis

**Figure 3:**
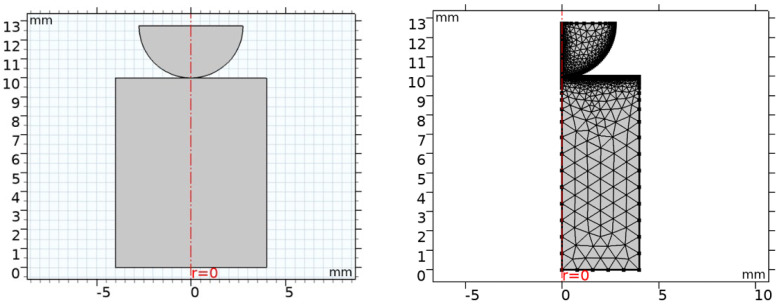
Two-dimensional axisymmetric finite element model of the compressive loading setup. (**Left**) Geometry of the cylindrical specimen (radius 4 mm, height 10 mm) and the bottom part of the spherical loading head (radius: 2.75 mm). The axis symmetry runs along the centre, marked with the red dashed line. (**Right**) Finite element mesh with triangular elements, featuring the refined meshing at the contact region gradually coarsening towards the bulk.

Finite element analysis (FEA) was performed using COMSOL Multiphysics (version: 5.5) to simulate the compressive loading conditions and validate the experimental results. A 2D axisymmetric model was designed based on the experimental setup. The cylindrical cement specimens loaded by a spherical indenter possess a rotational symmetry. The stress and displacement fields are also symmetric around the central axis, allowing the axisymmetric formulation to accurately reproduce the three-dimensional mechanical response while greatly reducing computational complexity. The cylindrical specimen (radius: 4 mm, height: 10 mm) and the spherical loading head (radius: 2.75 mm) were created in a 2D plain, see Figure 3. The mechanical parameters of the dental cements and loading head can be found in Table 1 which were based on the current literature [10,29,30,31,32].

Contact between the spherical loading head and specimen was modelled using contact pairs with a penalty formulation, implemented within an assembly defining the contact boundaries. A user-controlled mesh was applied, consisting of triangular elements. The maximum element size was set to 0.1 mm on the loading head surface and 0.05 mm on the surface of the specimen at the contact region, with a gradually coarsening mesh towards the bulk. This resulted in 879 mesh vertices, 1551 triangles, 203 edge elements, and 7 vertex elements. Mesh convergence was confirmed by repeating the simulations with increasingly refined meshes in the contact region, which produced negligible changes in peak stress values and load–displacement behaviour, verifying that the chosen mesh density was sufficient for accurate computation.

**Figure 4:**
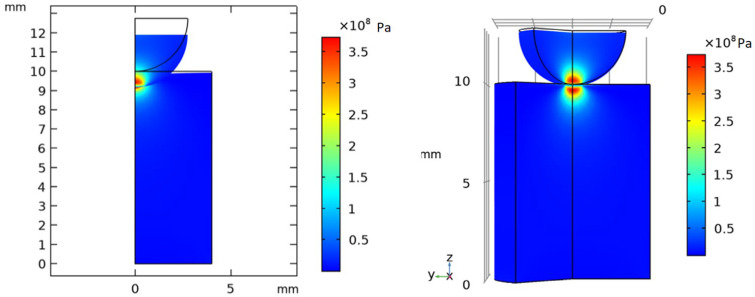
Von Mises stress distribution in the AphC specimen under compressive loading at 30 s, corresponding to the peak experimental force of 315 N. (**Left**) 2D axisymmetric finite element model showing the cylindrical specimen and spherical loading head, with the true calculated displacement (scale factor = 1). The maximum stress is observed at the contact region. (**Right**) 3D visualization generated by rotating the 2D axisymmetric results around the symmetry axis. A slice is removed from this representation to illustrate the internal mechanical stress distribution. The colour scale represents stress in Pa, with red indicating higher stress and blue indicating lower stress.

A time-dependent mechanical study was performed to simulate the static loading and unloading phases, using a triangular function over 60 s (30 s loading and 30 s unloading), reflecting the experimental protocol. The maximum mechanical stress was observed at 30 s. The simulation was solved using the MUMPS solver with a backward differentiation formula (BDF) method for time stepping. A representative von Mises stress distribution at 30 s is shown in Figure 4. The results are displayed using the 2D model with true displacements (scale factor = 1) and a 3D reconstruction generated by revolving the 2D solution. For visualization purposes, a slice of the 3D model was removed to reveal the stress distribution within the structure. In the results section, the displacement is scaled down for better visualization purposes. Mechanical stress and strain fields, as well as contact pressure, were analyzed to identify critical conditions corresponding to the experimental failure loads. The FEA predictions exhibited close correspondence with the experimental measurements, thereby confirming the validity of the numerical model.

### 2.4. Statistical Evaulation

The measured data were statistically analyzed with SPSS (SPSS version 23, IBM Corp., Somers, NJ, USA) and R (version 4.5.1; R Foundation for Statistical Computing, Vienna, Austria). Normality of data distribution was assessed using the Shapiro–Wilk test and homogeneity of variances was tested with Levene’s test. ANOVA and Tukey HSD tests were used to compare the mean values of different groups. The significance level was set to 5% (*p* < 0.05). The results are shown as mean value ± standard error of the mean. Kaplan–Meier survival analysis was used to estimate the survival probabilities of each group for the cycling dynamic tests. The (Breslow and weighted Peto-Peto) log-rank test was applied to compare the survival distributions across the groups, assessing statistically significant differences in fatigue performance.

## 3. Results

### 3.1. Results of the Static Loading Tests

**Figure 5:**
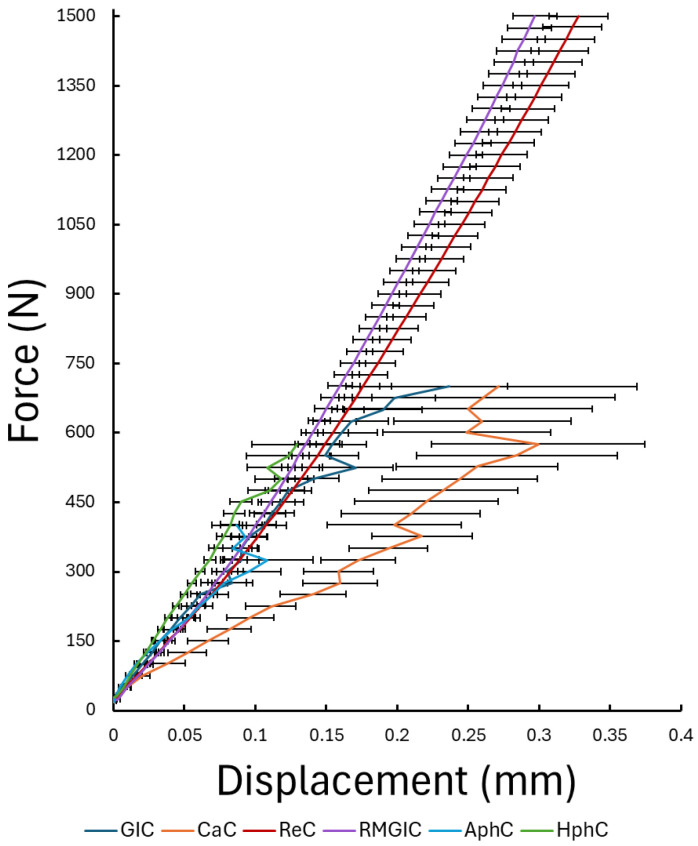
Force–average displacement curves for the tested six dental cements, obtained from static compressive loading tests. Each curve represents the mean response of the respective group, and the error bars indicate the standard error of the mean.

During the static compression tests on the dental cement samples, the displacement of the loading head was measured in response to the predefined, gradually increased loading force. In Figure 5, the force–mean displacement curves of each cement group can be seen. The results indicate that GIC, CaC, and phosphate cements exhibited similar mechanical performance, fracturing at a lower loading force compared to RMGIC and ReC. Additionally, the slopes of the curves are similar except the CaC, suggesting similar elastic response. The ReC and RMGIC samples remained intact under the maximum applied load, showing much higher mechanical strength.

**Figure 6:**
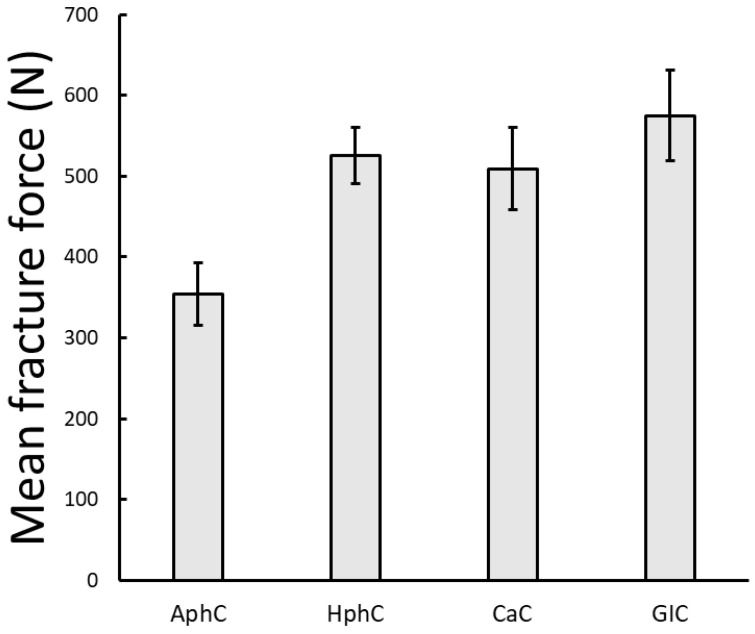
Mean fracture force values measured during static loading tests for different cement types. The error bars represent the standard error of the mean. (Note that ReC and RMGIC samples did not fracture during the static tests, and therefore they do not appear in this Figure).

Among the four weaker dental cements, the GIC exhibited the highest resistance to fracture, with a maximum of 575 ± 56 N mean force, while the AhpC showed the lowest resistance, with 354 ± 136 N mean fracture force. Fracture force values for RMGIC and ReC resin cements exceeded the maximum 1500 N, used in the static load protocol. One-way ANOVA statistical analysis of the measured maximum fracture force revealed significant difference between the different cement groups (*p* = 0.012 *). The fracture force values below 1500 N are shown in Figure 6 with the standard error values. It can be seen that the AphC cements fractured at significantly lower force values on average compared to the other cement types.

The results of the Tukey post hoc test are shown in Table 2. As RMGIC and ReC cements did not fracture under the test conditions, they were excluded from the statistical comparison in Table 2. The post hoc tests showed that AphC had a significantly different mean value compared to GIC (*p* = 0.009 *) and showed no statistical difference compared to the other phosphate cements HphC (*p* = 0.059) and CaC (*p* = 0.103). The post hoc test results also showed that there was no significant difference in the average fracture mean forces between the CaC and GIC (*p* = 0.744), CaC and HphC (*p* = 0.994), and GIC and HphC (*p* = 0.872).

**Figure 7:**
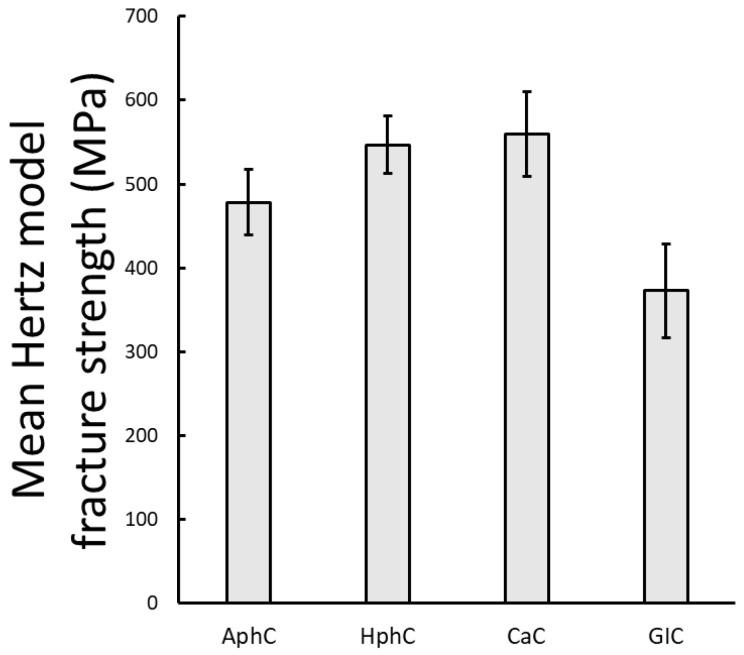
Mean compressive strength values calculated from static loading test results for each cement type using the Hertzian model for spherical loading head. The error bars represent the standard error of the mean.

The results of the mean strength were calculated from the average measured force values and the measured cross-sectional area of the samples based on the Hertzian formula (1) as described in the Methods section using the material constants given in Table 1. Hertzian contact mechanics has been applied to estimate the stresses generated by the spherical loading head. This model is appropriate for brittle dental cements since these materials deform elastically up to fracture and show negligible plasticity. Under these conditions, Hertzian theory provides an accurate approximation of the contact area and the resulting compressive stress at failure. The mean strength values can be seen in Figure 7. Among these dental luting cements CaC had the highest mean strength. The modulus of elasticity value is included in the Hertzian contact mechanics model and shifts the mechanical stress and strength values compared to the mean maximum force, thus changing the previous order.

### 3.2. Results of the Dynamic Loading Tests

The accelerated dynamic loading tests provided insight into the fatigue behaviour of the dental luting cements. The median and average number of survival cycles, shown in Table 3, was calculated for each cement group based on the number of cycles completed before the failure of a sample.

One-way ANOVA revealed significant differences in the number of survival cycles between the dental cement groups (*p* < 0.001 *). The subsequent Tukey HSD post hoc test results, presented in Table 4, showed a pattern consistent with the static loading test: RMGICs and ReCs exhibited no significant difference in mean survival cycles (*p* = 0.97), with most samples surviving the entire protocol. In comparison, the other cements had significantly lower average survival cycles values (*p* < 0.05). Among GIC, CaC, AhpC, and HphC, no significant differences were observed (*p* > 0.05).

The dynamic load tests were carried out across seven different loading phases in order to evaluate fatigue behaviour under different loading ranges. A total of 10,500 cycles were distributed across these phases. The number of fractures that occurred in each phase for the cement groups is shown in Table 5, which illustrates the distribution of failures across the dynamic loading protocol.

**Figure 8:**
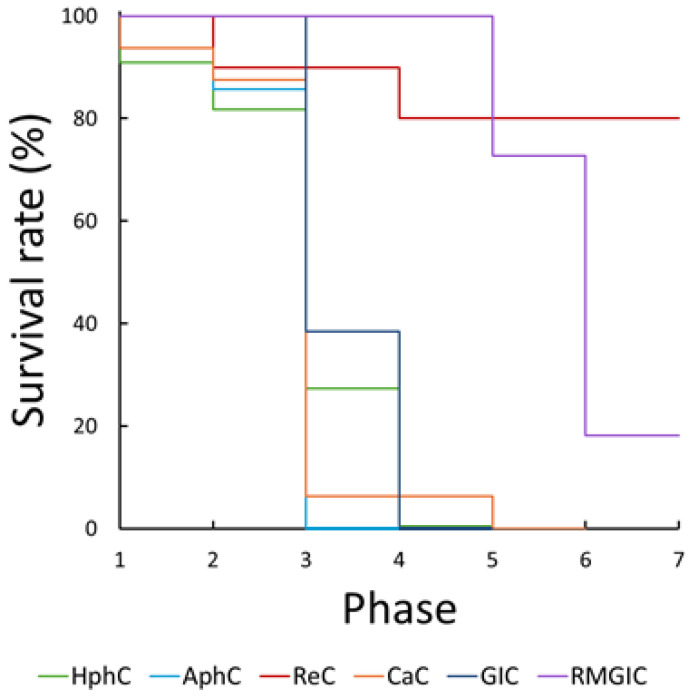
Kaplan–Meier survival curves showing the dynamic fatigue performance of the tested dental cements under cyclic loading.

Additionally, a survival analysis was applied to visualize and compare the fatigue resistance among cements. Kaplan–Meier survival analysis was performed to assess the survival probability of each cement group over the dynamic loading test. The resulting survival curves, presented in Figure 8, demonstrate that resin cement samples exhibited the highest survival probability, followed by RMGIC, and by the weaker cements (GIC, CaC, AhpC, and phosphate cements) which showed lower survival probabilities.

To further compare the survival distributions of the dental cements, log-rank tests (Breslow) were performed based on Kaplan–Meier survival estimates. The pairwise comparisons revealed significant differences in fatigue survival among the cement types as shown in Table 6. Significant differences were observed between RMGIC and ReC compared to all other cement types, while the RMGIC and ReC showed statistically no significant difference (*p* = 0.096), indicating higher fatigue resistance. The GIC showed a significant difference compared to AphC (*p* = 0.030 *) and CaC (*p* = 0.030 *), while the AphC, HphC, and CaC did not show statistically significant differences in their fatigue resistance properties. These findings suggest that RMGIC and resin cements outperform phosphate, carboxylate, and GICs in fatigue resistance both under lower and higher loading conditions. As the survival curves indicated non-proportional hazards, an additional Peto-Peto weighted log-rank test was performed. This analysis produced only minor numerical changes relative to the standard Breslow log-rank test, and all substantive conclusions regarding differences in fatigue survival between cement groups remained unchanged.

### 3.3. Results of Finite Element Analysis

**Figure 9:**
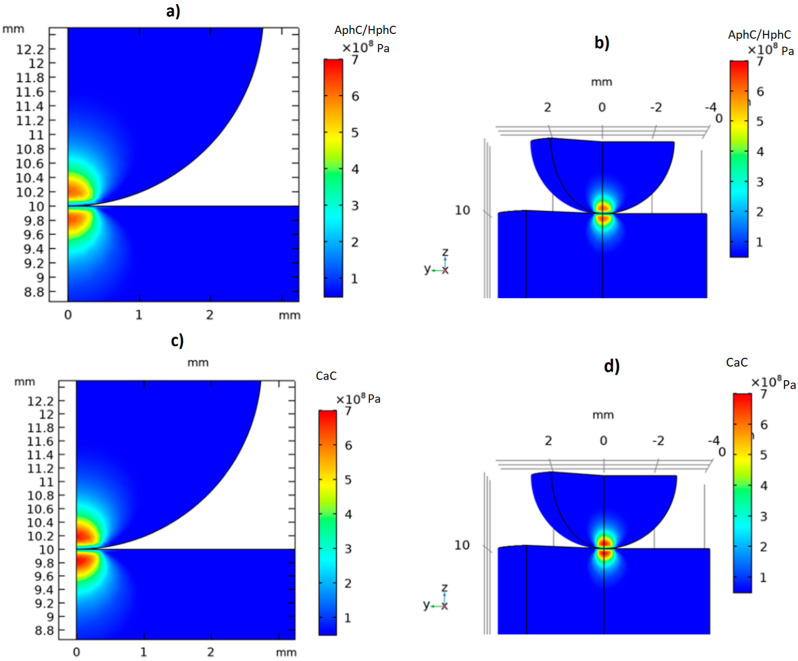

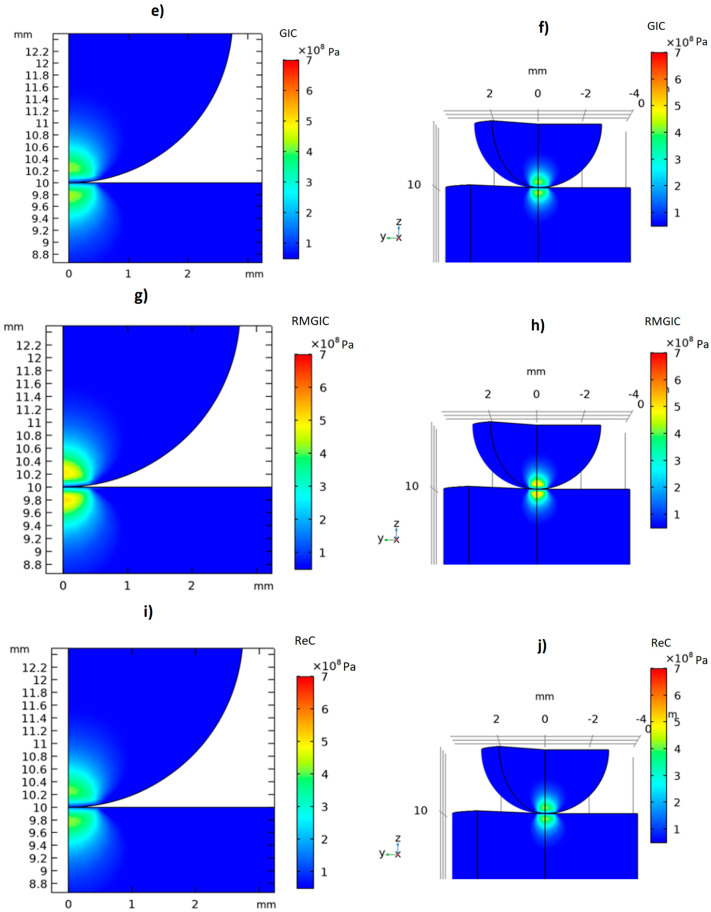
Finite element analysis of dental luting cements: 2D and rotated 3D models showing von Mises stress distribution (Pa) under static loading with a spherical loading head for (**a**,**b**) showing phosphate cement, (**c**,**d**) carboxylate, (**e**,**f**) GIC, (**g**,**h**) RMGIC, (**i**,**j**) self-adhesive resin cement (ReC). The colour scale represents stress in Pa unit.

Finite element modelling was employed to analyze the mechanical stress distribution in the dental luting cements under the static loading protocol with a spherical indenter loading head. The simulations revealed the stress patterns across the cement types, as illustrated in Figure 9. In the case of the phosphate and carboxylate cements, higher von Mises stress concentrations were observed near the contact area of the spherical loading head, indicating a greater susceptibility to localized failure under static loads. The maximum von Mises stress reached 600–700 MPa in these cases when applying 350–500 N compression force. In contrast, the GIC, RMGIC, and ReC exhibited much lower mechanical stress concentrations and a more uniform distribution, with a maximum of 300–400 MPa von Mises stress. Our finite element method results also suggest that the mechanical durability of the GIC, RMGIC, and ReC may be attributed to their ability to distribute mechanical stress more effectively.

## 4. Discussion

This study comprehensively evaluated the mechanical behaviour of six commonly used definitive dental luting agents under static and dynamic loading conditions, supported by finite element analysis. The findings clearly indicate that resin-based and resin-modified glass ionomer cements (ReC and RMGIC) outperform conventional aqueous-based cements, including zinc phosphate, polycarboxylate, and traditional glass ionomer cements, in both compressive strength and fatigue resistance. These results have important clinical implications, particularly for the selection of luting agents in restorations exposed to high mechanical loads, such as fixed prosthodontics.

### 4.1. Static Load Performance

Under static compressive loading, ReC and RMGIC samples demonstrated superior resistance, showing no fracture even under the maximum applied force of 1500 N. In contrast, conventional cements (AphC, HphC, CaC, and GIC) fractured at considerably lower forces. Among these, GIC showed a relatively higher mean fracture load, while AphC exhibited the lowest. These results are consistent with earlier reports indicating that resin-based materials offer enhanced mechanical strength due to their polymeric matrix and improved filler content [19]. The observed low fracture resistance and brittleness of conventional zinc phosphate cement (AphC) align with its well documented mechanical limitations [1,2,10,34,35]. The application of the Hertzian contact model enabled a more accurate compressive stress estimation by accounting for material elasticity, Poisson’s ratio and geometry. As a result, cements with higher stiffness like CaC showed elevated mechanical stress values despite comparable fracture forces, demonstrating the importance of elastic properties in interpreting the strength of the materials. Interestingly, CaC exhibited relatively higher compressive strength than GIC, likely reflecting its viscoelastic behaviour and potential for a limited chemical bonding structure. However, its overall reliability under high load remains inferior to resin-based materials. These findings reinforce the concept that materials with greater stiffness concentrate local stresses more strongly under spherical indentation [1,3,25,28]. The statistical comparisons, including ANOVA and Tukey post hoc tests, confirmed significant intergroup differences, particularly the lower performance of AphC relative to GIC, highlighting the role of composition and powder-to-liquid ratio variations between phosphate-based formulations [11,13,16].

If the mechanical stress exceeds the strength of the material, it will fracture [10,36,37,38,39,40]. In general, dental cements have low elasticity and almost no plasticity at all. Before fracturing, microcracks form in the structure of the material. Microcracks lead to fatigue weakening of the materials and result in fracturing the materials at lower stress values than their strength. This causes a large number of fixed replacements to fail and can even lead to displacements in the restorations [10]. The fragmentation of the fixing cement can later lead to the development of microbial infection [11]. High compressive strength provides a greater resistance to the mastication forces, thereby increasing the firmness of the restoration and the resistance to external influences [36].

Yli-Urpo et al. studied the mechanical properties of GICs and a special bioactive glass particle reinforced cement [39]. They fabricated similar specimens as shown in our study and found that the compressive strength might be weakened using the bioactive glass reinforcement phase in the GIC.

### 4.2. Fatigue Behaviour and Clinical Relevance

The repeated mastication movements can be modelled by dynamic, cyclic loading of the material, as they can reproduce the dynamically changing forces that occur during normal mastication. The results can therefore be related to clinical situations [40,41]. In practice, it is rare for a restoration to fail immediately after the restoration process, whereas fatigue fracture due to cyclic force is one of the most common causes of clinical failure [19].

Dynamic loading testing provides a closer approximation of the cyclic masticatory forces acting in vivo, offering valuable information beyond static failure points. Here again, resin-based (ReC) and resin-modified (RMGIC) cements outperformed the conventional cements, exhibiting the highest fatigue resistance, with most samples surviving all loading phases without failure. Their improved fatigue resistance may be attributed to both their intrinsic toughness and ability to dissipate cyclic stresses via viscoelastic deformation due to their resin-modified structure [3,11,19]. In contrast, phosphate and polycarboxylate cements demonstrated markedly reduced fatigue resistance, with failures commonly occurring during mid-level loading phases (400–800 N). Kaplan–Meier survival analysis and log-rank testing confirmed statistically significant differences between resin-based and conventional materials. Notably, GIC showed moderate performance, offering a compromise between mechanical strength and biological benefits such as fluoride release and biocompatibility [3,11,16]. The fatigue resistance patterns during the accelerated dynamic loading tests were consistent with the static results: most of the ReC and RMGIC samples maintained structural integrity throughout all 10,500 cycles, indicating excellent fatigue performance. In contrast, conventional cements failed at earlier stages, especially during higher loading phases (800–1600 N). These results support the clinical recommendation of resin-based materials in high-stress restorations for long-span prostheses, or cases with limited cement thickness [19,27]. Resin-based cements (ReC and RMGIC) exhibited significantly higher survival probabilities compared to aqueous-based groups, which is in line with the findings of other studies, emphasizing the enhanced durability and cohesive strength of resin-based systems [3,19]. Notably, no significant differences were found between GIC, CaC, and phosphate cements, suggesting a general mechanical limitation shared across traditional cement types.

### 4.3. Finite Element Analysis

The finite element simulations supported the experimental results by revealing distinct stress distributions among the different materials. Conventional cements (AphC, HphC, CaC) exhibited high localized stress concentrations at the contact region, with von Mises stress values exceeding 600 MPa at moderate loading levels. This behaviour explains their lower fracture resistance and susceptibility to microcrack initiation leading to earlier mechanical failure and brittle fracture. Conversely, RMGIC and ReC showed lower peak stresses (300–400 MPa) and more homogenous stress distributions, indicating efficient stress dissipation and improved mechanical stability. Their greater contact compliance with the loading head likely reduces stress concentration, thereby enhancing fatigue resistance. These results are consistent with previous computational analysis of composite and viscoelastic dental materials [28,32]. The strong correlation between the experimental and simulated results validates the Hertzian-based contact mechanical model as a reliable approach for evaluating cement behaviour. The pronounced, localized stress concentrations observed in the phosphate and polycarboxylate cements reflect their inherent brittleness and limited capacity for stress redistribution, making them more susceptible to microcrack initiation. By contrast, the more diffuse stress fields and lower peak stresses in RMGIC and resin cements are consistent with their viscoelastic or polymer-reinforced structure, which enables more effective dissipation of mechanical loads. Future studies may further enhance this model by incorporating microstructural heterogeneity and viscoelasticity, time-dependent material degradation, and temperature or moisture (hydrothermal) effects, thereby offering a more clinically relevant simulation environment. Several recent publications explore the mechanical properties of luting materials, including the biomechanical behaviour of resin cement and the viscoelastic behaviour of aged resin-based dental composites [23,42,43,44,45]. Topics covered include the finite element analysis of resin cement with different elastic moduli and investigations into viscoelastic deformation mechanisms [42,43,45]. The FEA simulations effectively validated the experimental results, revealing consistent von Mises stress distributions across cement types under simulated loading conditions. Stress concentrations in phosphate and carboxylate cements were more localized near the indenter interface, indicating a higher risk for crack initiation and propagation. In contrast, the more uniform stress distribution in resin and RMGICs suggests their capacity to manage applied loads more efficiently, reducing the likelihood of failure [22,24]. The maximum simulated stresses for weaker cements exceeded 600 MPa at failure loads, correlating well with experimentally determined fracture points. These findings demonstrate the utility of FEA not only as a tool for validating physical experiments but also as a predictive method for assessing material behaviour under complex loading scenarios.

Although the use of resin-based cements is becoming more widespread in everyday practice, conventional cements, e.g., zinc phosphate, still have indications mainly in non-esthetic, low-stress bearing areas. Its advantages include a long history of use and relatively low cost. Polycarboxylates are mainly used where chemical adhesion to both dentin and enamel is desired and for sensitive teeth because their pH is less acidic compared to zinc phosphate. While polycarboxylates’ mechanical strength and solubility in oral fluids are inferior to zinc phosphate and modern resin cements, their use remains appropriate as an intermediate restorative material or base/liner in routine clinical practice regarding their fluoride release ability [8,24,46].

### 4.4. Limitations

Although the study design aimed to replicate clinically relevant loading conditions, certain limitations should be acknowledged. The oral environment presents significant limitations for all dental cements due to variables like temperature fluctuations and continuous moisture exposure. These factors can compromise their mechanical integrity and fatigue resistance over time. Conversely, while resin-based cements offer superior mechanical stability and lower water solubility, they are not completely resistant; temperature changes can accelerate the degradation of the resin matrix or affect the stability of the adhesive interface with the tooth structure, ultimately limiting the long-term durability of restorations when compared to laboratory ideal conditions [23,24,43]. The test setup used simplified geometries and loading protocols, which may not fully capture the variability of intraoral environments, such as temperature fluctuations, moisture, pH fluctuations, or complex uneven occlusal stress patterns. In addition, although the sample size was statistically adequate, larger datasets and long-term ageing or in vivo validation would further improve reliability. Future investigations should integrate these factors to extend the clinical relevance of the results. These investigations could focus more on the thermocycling and long-term water ageing to better replicate intraoral degradation, and may also extend the finite element model to full 3D simulations in cases where asymmetric geometries or loading conditions become clinically relevant.

## 5. Conclusions

This study provided a systematic comparison of six widely used dental luting cements under both static compressive loading, dynamic fatigue, and finite element analysis. The results demonstrate clear performance differences between resin-based and conventional aqueous-based cements, with significant implications for clinical practice.

The key findings are summarized as follows:

- Resin-based (ReC) and resin-modified glass ionomer (RMGIC) cements exhibited superior mechanical strength and fatigue resistance, showing no fractures even at maximum test conditions. Their performance indicates excellent long-term reliability, making them the materials of choice for high-stress clinical situations such as full-coverage crowns, long-span bridges, or implant-supported restorations.
- In contrast, conventional cements, such as glass ionomer (GIC), polycarboxylate (CaC), and zinc phosphate cements (AphC, HphC) displayed lower compressive and cyclic load resistance. Among these, GIC showed relatively better static performance, while zinc phosphate cement exhibited the lowest mechanical strength. Despite their limitations, these traditional materials may still be suitable for low-stress, short-span, or temporary applications where chemical adhesion or fluoride release are desired advantages.
- Finite element analysis confirmed that resin-based and resin-modified cements distribute stress more evenly under load, reducing the risk of crack initiation and explaining their enhanced mechanical durability. The agreement between experimental and computational results validates the applied testing methodology.
- Clinically, understanding the mechanical and structural behaviour of luting agents is crucial for selecting the most appropriate material and ensuring the longevity of fixed restorations. Future studies should address long-term degradation under thermocycling and humidity to further approximate the intraoral environment.

## Figures and Tables

**Table 1:** Mechanical parameters used in the calculations and FEA study [10,29,30,31,32].

Material	Density (kg/m^3^)	Modulus of Elasticity (GPa)	Poisson’s Ratio
AhpC	3250	13.5	0.3
HphC	3250	13.5	0.3
CaC	2570	16	0.3
GIC	1880	7.7	0.3
RMGIC	1880	9.9	0.3
ReC	1700	7.4	0.3
Loading head	7850	220	0.3

**Table 2:** Results of the Tukey post hoc test comparing the averaged maximal measured force values for fracturing among the dental cement groups. The analysis includes the four types of dental luting cements that exhibited fracture lower than the maximal 1500 N static load.

*p* Values	GIC	HphC	CaC	AhpC
GIC	1	0.872	0.744	0.009 *
HphC		1	0.994	0.059
CaC			1	0.103
AhpC				1

* shows significant difference.

**Table 3:** Median number of survival cycles during mechanical fatigue testing (mean ± SEM in parentheses).

	HphC	AphC	ReC	CaC	GIC	RMGIC
Survival cycle number	4567 (4785 ± 341)	4507 (4303 ± 207)	10,500 (9489 ± 706)	4505 (4663 ± 273)	5295 (5399 ± 206)	9025 (9070 ± 291)

**Table 4:** Results of the Tukey HSD post hoc test comparing the survival cycle numbers between the different dental cements during the fatigue loading tests.

	HphC	AphC	ReC	CaC	GIC	RMGIC
HphC	1	0.965	<0.001 *	0.99	0.829	<0.001 *
AphC		1	<0.001 *	0.987	0.417	<0.001 *
ReC			1	<0.001 *	<0.001 *	0.97
CaC				1	0.606	<0.001 *
GIC					1	<0.001 *
RMGIC						1

* shows significant difference.

**Table 5:** Number of fractures per cement group over seven loading phases in the dynamic loading test (10,500 cycles total).

	Phase 1	Phase 2	Phase 3	Phase 4	Phase 5	Phase 6	Phase 7	No Fracture
HphC	0	1	1	6	3	0	0	0
AphC	0	0	1	6	0	0	0	0
ReC	0	0	1	0	1	0	0	8
CaC	0	1	1	13	0	1	0	0
GIC	0	0	0	8	5	0	0	0
RMGIC	0	0	0	0	0	3	6	2

**Table 6:** Resulting *p* values of the log-rank tests comparing survival distributions of dental cement types.

	AphC	HphC	CaC	GIC	RMGIC	ReC
AphC	1	0.452	0.725	0.030 *	<0.001 *	0.004 *
HphC		1	0.540	0.285	<0.001 *	0.002 *
CaC			1	0.030 *	<0.001 *	<0.001 *
GIC				1	<0.001 *	0.002 *
RMGIC					1	0.096
ReC						1

* shows significant difference.

## Data Availability

The original raw data contributions presented in this study are included in the article and Supplementary Materials. Further inquiries can be directed to the corresponding author.

## Supplementary Materials

The supporting information can be downloaded at https://www.mdpi.com/article/10.3390/dj13120601/s1. File S1: The supplementary file contains the raw data of the measurements used for the analyses presented in this study.

## Abbreviations

The following abbreviations are used in this manuscript:

AphC	Adhesor Zinc Phosphate Cement
HphC	Harvard Zinc Phosphate Cement
CaC	Harvard Polycarboxylate Cement
GIC	Glass Ionomer Cement
RMGIC	Reinforced Glass Ionomer Luting Cement
ReC	Self-Adhesive Resin Cement
